# Resin infiltration or bioactive adhesive: which treatment provides better esthetic satisfaction for post-orthodontic white spot lesions?

**DOI:** 10.1038/s41432-026-01241-z

**Published:** 2026-07-24

**Authors:** Hisham Mohammed, Matthew Nangle, William Aung, Deborah Du

**Affiliations:** Discipline of Orthodontics, School of Dentistry, the University of Queensland, Brisbane, QLD Australia

**Keywords:** Dental biomaterials, Fixed appliances

## Abstract

**A Commentary on:**

**Albashaireh ZS, Altallaq MK**.

Assessment of patient satisfaction and esthetic outcomes following treatment of post-orthodontic white spot lesions using a bioactive adhesive and resin infiltrant: a randomized split-mouth clinical trial. *Journal of Dentistry*. 2026;167:106565.

**Design:**

Split-mouth randomized clinical trial of 20 orthodontically treated patients (13 female, 7 male) involving 120 teeth with anterior white spot lesions (WSLs). Each participant received ICON resin infiltration or Hi-Bond Universal in contralateral quadrants. Esthetic changes were assessed by the patients and by two independent clinicians using a 100-point visual analogue scale (VAS) at one-month recall.

**Case selection:**

Participants aged 16–30 years and who had completed fixed orthodontic treatment at least one month before enrollment were included. Eligible participants presented with at least one visible WSL on the labial surfaces of the anterior teeth or premolars on contralateral quadrants. All participants were required to have good oral hygiene, clinically healthy gingival tissues, and no evidence of active periodontal disease.

**Data analysis:**

Categorical outcome data were presented using descriptive summary measures including frequencies and percentages. The proportion of satisfactory vs. unsatisfactory outcomes (paired binary data) was compared between the Hi-Bond Universal and ICON treatments using the McNemar test.

**Results:**

Overall patient satisfaction was equally high (80%) for both treatments, and there were no significant differences between patient or clinician ratings. Inter-assessor agreement was deemed “fair” for both groups (Cohen’s kappa 0.318–0.340). Thus, both ICON and Hi-Bond Universal effectively masked WSLs, as deemed by subjective esthetic evaluation.

**Conclusion:**

Both bioactive adhesives and resin infiltration achieve satisfactory esthetic outcomes in masking demineralized enamel, at least in the short-term. Further longitudinal studies comparing the outcomes of these materials would be beneficial to determine whether these effects are sustained over time.

## GRADE Rating:



## Commentary

White spot lesions (WSLs) are a common adverse outcome following fixed orthodontic therapy and can compromise dental esthetics. The prevalence of WSLs after orthodontic treatment is 2–97%^1^. Minimally invasive treatments such as resin infiltration reinforce areas of demineralization, reduce the risk of further decay, and improve the appearance of affected teeth^2,3^. However, due to its high cost, alternatives such as bioactive glass adhesives have been considered for esthetic masking of WSLs, as they may offer a simpler and potentially more cost-effective option.

This study^4^ is a split-mouth randomized clinical trial of 20 orthodontically treated patients with anterior WSLs (*n* = 120 teeth). Each participant received ICON or Hi-Bond Universal in contralateral quadrants. Patients and two calibrated clinicians independently assessed esthetic changes using a 100-point visual analogue scale (VAS), with scores categorized into five ordinal ranks: Slight (0–20 mm), Mild (21–40 mm), Moderate (41–60 mm), High (61–80 mm), and Outstanding (81–100 mm). These scores were further dichotomized into satisfactory (High and Outstanding; 61–100 mm) and unsatisfactory (Moderate, Mild, and Slight; 0–60 mm) outcomes. Overall, patient satisfaction was similarly high for both treatments (80%), with no significant differences identified between patient or clinician ratings.

A computer-generated randomization sequence was used, and a secondary person assigned the interventions to the quadrants. All participants completed follow-up assessment; therefore, the risk of attrition bias was low. Selective reporting could not be ascertained, as the full study protocol was not published. The study followed Consolidated Standards of Reporting Trials (CONSORT) guidelines and received ethics approval before participant recruitment.

The split-mouth design was appropriate as it controlled for patient-level confounders such as oral hygiene, diet, oral function, salivary factors, and any systemic influences. To minimize crossover effects during material application, rubber dam isolation was used. Baseline lesion severity was balanced between contralateral quadrants. A double-blind design was used with both patients and clinical assessors blinded to treatment allocation. Although the sample size (*n* = 120 teeth) was reportedly powered for the primary outcome, this did not match the initial estimated sample size provided on the trial registry website (*n* = 150). Additionally, clustering effects associated with the split-mouth design may not have been fully accounted for. As demonstrated by Mheissen et al., sample size calculations in split-mouth design should be adjusted based on the number of teeth measured per patient and the intra-cluster correlation coefficient (ICC)^5^. Failure to account for these factors may have resulted in an underpowered split-mouth clinical trial.

The study addressed a clinically relevant question in an appropriate target population, and both patient-reported and clinician-reported assessments were sought for evaluating esthetic outcomes. The choice of ICON resin infiltrant represented an appropriate gold-standard comparator for the bioactive adhesive. However, several methodological limitations should be considered. Esthetic judgments relied solely on standardized DSLR photographs viewed on a calibrated monitor. More objective methods such as quantitative light-induced fluorescence or spectrophotometric analysis may have provided greater objectivity.

Although VAS scoring is commonly used in esthetic judgments, its subjective nature remains a limitation. In addition, categorization and dichotomization of VAS scores may have reduced statistical sensitivity. Inter-assessor agreement was only “fair” for both treatment groups (Cohen’s kappa 0.318–0.340).

Generalizability is limited by the single-center design, small sample size, predominantly young patient population, and short follow-up duration. Most participants were adolescents or young adults recruited from a single clinic setting. Additionally, exclusion of patients with poor oral hygiene or periodontal disease may reduce applicability to broader orthodontic populations, where larger or more extensive WSLs may occur more frequently.

Overall, the study was judged to provide low-quality evidence. Despite its randomized design, the certainty of the evidence was downgraded because of imprecision resulting from the small sample size, uncertainty regarding reporting bias, and limited external validity arising from the single-center design and short follow-up period. In summary, the study provides preliminary evidence that bioactive adhesives may offer a clinically acceptable alternative to resin infiltration for short-term masking of post-orthodontic WSLs. However, larger studies with longer follow-up periods and more objective outcome assessments are required.
